# Statistical evaluation of methods for quantifying gene expression by autoradiography in histological sections

**DOI:** 10.1186/1471-2202-10-5

**Published:** 2009-01-15

**Authors:** Stanley E Lazic

**Affiliations:** 1 Cambridge Computational Biology Institute, Department of Applied Mathematics and Theoretical Physics, University of Cambridge, Cambridge, CB3 0WA, UK

## Abstract

**Background:**

In situ hybridisation (ISH) combined with autoradiography is a standard method of measuring the amount of gene expression in histological sections, but the methods used to quantify gene expression in the resulting digital images vary greatly between studies and can potentially give conflicting results.

**Results:**

The present study examines commonly used methods for analysing ISH images and demonstrates that these methods are not optimal. Image segmentation based on thresholding can be subject to floor-effects and lead to biased results. In addition, including the area of the structure or region of interest in the calculation of gene expression can lead to a large loss of precision and can also introduce bias. Finally, converting grey level pixel intensities to optical densities or units of radioactivity is unnecessary for most applications and can lead to data with poor statistical properties. A modification of an existing method for selecting the structure or region of interest is introduced which performs better than alternative methods in terms of bias and precision.

**Conclusion:**

Based on these results, suggestions are made to reduce bias, increase precision, and ultimately provide more meaningful results of gene expression data.

## Background

In situ hybridisation has been used as a standard method for quantifying gene expression in histological sections for nearly forty years. Oligonucleotide or RNA probes are either labelled with radioactive atoms such as ^35^S or ^32^P, or other non-radioactive molecules such as biotin. The location of the bound probe in the tissue is then usually visualised by autoradiography or immunohistochemistry, and captured as a digital image for quantification. Images of autoradiographic films are analysed in a semiquantitative manner where the darkness of the film is proportional to the amount of gene expression. This method is semiquantitative because the darkness of the film is only proportional to the amount of gene expression, but there is no way to map the darkness to the number of transcripts. This paper focuses on the analysis of autoradiographic films from in situ hybridisations, but the results should generalise to other autoradiographic methods such as 2-fluoro-deoxyglucose [1].

The methods used to quantify the amount of gene expression in the resulting images vary widely between studies and laboratories, and could potentially give conflicting results. There are two main steps in the analysis where variations in methods arise. The first is during image segmentation, or the process of determining what is to be included in the analysis (foreground) and the rest (background). During segmentation, setting a threshold based on pixel intensity is a common method, but the choice of cut-off value varies from 2 to 3.5 standard deviations above the mean background level [2,3]; alternatively, the threshold may be manually adjusted [4]. More sophisticated thresholding methods using Bayesian classification have also been developed [5], and there are some forty algorithms to automatically threshold images [6], although only a few are actually used in the neuroscience literature. Alternative methods for segmenting the image include outlining the structure by hand [7-11] or using a magic want tool [12], typically in combination with subtracting background levels. To help identify the boundaries of the structure when outlining by hand, an image of the film and another of the stained tissue can be superimposed [13]. Another method involves using a 'template' or standard sized selection window which is constant across all sections and animals, and which is placed over the area of highest intensity in the structure of interest [14]. For example, Van Hoomissen and colleagues placed an 8 × 10 pixel oval over the locus coeruleus [15]. In this study, analysis of hippocampal subregions also involved placing ten 8 × 8 pixel ovals in random locations and taking an average. An unusual method by Gartside et al. involved placing lines of 100 pixels in length perpendicular to the dentate gyrus (DG), CA1, and CA3 subregions of the hippocampus, which sampled only a small part of the structures of interest and included many pixels in the analysis that were not part of the hippocampal subregion of interest [16].

The second step where variations in methods occur is in the processing of grey level (GL) values obtained from the digitised images. Once the foreground has been selected and measured, the resulting grey level values (typically 8-bit values from 0–255) are often converted into a variety of other units and expressed in various ways. This includes using one of several equations (see below) to convert the results to an optical density (OD). Sometimes GL values are multiplied by the area or the number of pixels of the structure or region of interest to give an 'integrated' value [17,18], sometimes they are divided by the area and expressed as intensity/mm^2 ^[7], sometimes the area is measured and reported as a separate variable [19], and sometimes the area is not included at all. In addition, many studies take into account the nonlinear nature of the film's response to radioactivity by using a ^14^C standard, and convert the GL values into units of radioactivity [7,20-23]. Many studies provide insufficient information to determine how the quantification was carried out, and analysis of the same dataset with various permutations of the above methods could potentially give different results. There is a need for standardisation of methods across studies, and a stronger theoretical underpinning for the methods used.

What is the best way to quantify gene expression in histological sections when analysing autoradiographic films? While there is likely no single method that will be superior in all cases, this paper analyses a set of images with a variety of methods in order to determine which method of segmentation produces the best results, defined in terms of greatest precision (as more precise estimates lead to greater statistical power) and potential for bias. In addition, a simulation study was used to determine the effects of various transformations of GL values. Other factors such as the time to carry out procedure are also considered. A modification of an existing method of segmentation is introduced which performs better than current commonly used methods.

## Methods

### Animals

Seventeen male Sprague-Dawley rats (Harlan, Oxon, UK) were housed individually and were eight weeks old at the start of the experiment. Ambient temperature was maintained at 21°C and humidity at 55% with ad libitum access to food and water. Animals were kept on a reversed 12-hour light/dark cycle (lights off at 10:00 AM). Animal experiments conformed to the UK Animals (Scientific Procedures) Act 1986, and procedures were carried out under appropriate Home Office (UK) project and personal licences.

### In situ hybridisation

Brains were sectioned at 20 *μ*m with a cryostat at -20°C, and every sixth section was placed onto polylysine-coated slides (Sigma, Dorset, UK). Three sections per animal were used, with the first section beginning at approximately -2.80 mm from the bregma [24]. Sections were allowed to air dry at room temperature and were then fixed with 4% paraformaldehyde for 5 min, washed in phosphate-buffered saline (PBS) and then dehydrated in 70% and 95% ethanol for 5 min before finally storing in fresh 95% ethanol. ISH was carried out under RNAase-free conditions and the mineralocorticoid receptor (MR) probe had the following sequence: 5' TTC GGA ATA GCA CCG GAA ACG CAG CTG ACG TTG ACA ATC T 3'. The probe was end-labelled with ^35^S and incubated at 37°C for one hour. The labelled probe was purified by centrifuging at 3000 rpm for two minutes through a G-50 sephadex micro-column (Amersham, UK).

Appropriate volumes of the labelled probe were added to hybridising buffer and the probes were evaluated for incorporation of the radiolabel by scintillation counting. Probes were hybridised overnight at 44°C and unbound probe was washed with saline sodium citrate (SSC; Sigma, UK) twice for 30 min at 55°C followed by 2 min washes with SSC, distilled water, 50%, 70% and 95% ethanol. Sections were allowed to dry at room temperature before exposure to the film.

^14^C-labelled standards of known radioactivity (range 30–862 nCi/g; Amersham) were placed in the X-ray cassette along with the brain sections and exposed to Kodak BioMax MR autoradiographic film (Amersham Biosciences) for six days. The film was developed with a Fuji Medical Film Processor (FPM-100A; Fuji Photo Film UK, London, UK).

### Image acquisition

The film was placed on a light box (Universal Electronics Industries, Hong Kong) and images recorded with a CCD camera (C3077; Hamamatsu) and Scion imaging software (Scion Corporation, Maryland, US). Images were 768 × 512 pixels and saved as 8-bit greyscale TIFF files. Figures were prepared with the GNU Image Manipulation Program (version 2.4.6).

### Image analysis and quantification

Images were analysed with the NIH ImageJ software (version 1.37; ). The expression of the MR receptor in the dentate gyrus of the hippocampus was quantified by determining the grey levels of the pixels using four different methods. The left and right side of the dentate gyrus was quantified separately on three sections and the background level on each section was measured and subtracted.

#### Method 1

**Figure 1:**
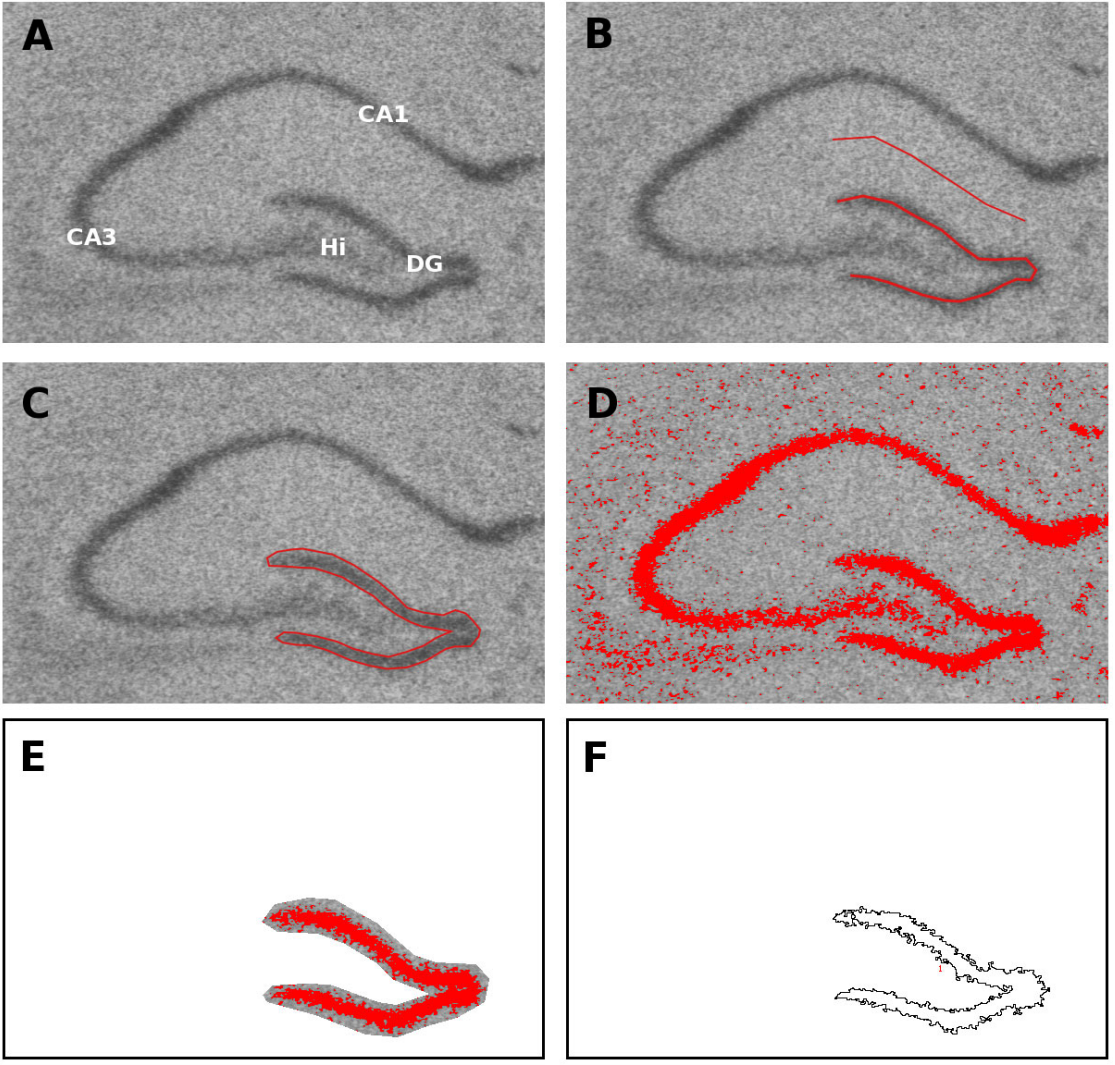
**Four methods of selecting the dentate gyrus**. The first method used a segmented line drawn down the centre of the DG (B). The background was measured with another line midway between the CA1 and the suprapyramidal blade of the DG. The second method used the polygon tool to outline the DG by hand (C). The third method used a thresholding approach and included only those parts of the DG that were three standard deviations above the mean background GL (D-F). The threshold was adjusted based on the calculated value (D) and then the DG was outlined and the background cleared in order to isolate the DG (E). The outline was only approximate as there was no need to distinguish between background and foreground precisely. The average GL and area were then calculated and the outline of the selected region is shown (F). The fourth method used a mixture model to determine the optimal threshold, and the rest of the analysis was carried out as above. CA = Cornu Ammonis, DG = Dentate gyrus, Hi = Hilus.

A segmented line was drawn down the centre of the DG (Fig. 1B) and the mean grey level was calculated. The background was measured as midway between the CA1 and the suprapyramidal blade of the DG using the same segmented line tool, and both the mean and standard deviation of the background recorded. There is nothing special about using a line, but in the case of the DG, a single line has the property of sampling the majority of the structure while staying away from the edges, which are imprecisely defined. Structures of other shapes can be segmented by 'outlining' them as in Method 2 (below), but staying well within the interior of the structure.

#### Method 2

The DG was outlined by hand using the polygon tool and the mean GL and the area were recorded (Fig. 1C).

#### Method 3

A thresholding approach was used which included only those parts of the dentate gyrus that were three standard deviations above the mean background GL (Fig 1D–F). After setting the threshold, the DG was outlined and the background cleared in order to isolate only the DG. The outline was only approximate as there was no need to distinguish between background and dentate gyrus precisely. The average GL and area were then calculated for the thresholded region. Only pixels in groups of 50 or greater were included, which eliminated stray pixels not a part of the DG but that were above the threshold.

#### Method 4

The DG was also selected using a mixture modelling approach (available as plugin for ImageJ). This is an automated thresholding method which fits two Gaussian distributions to the pixel intensity histogram of the whole image and sets the threshold at the intersection of the distributions. This is not a commonly used method for ISH analysis but was included for comparison because it is an automated procedure, which has the advantage of being fast, objective, and reproducible, and the results corresponded well to subjective visual estimates of the anatomical boundaries of the dentate gyrus.

#### Methods 5–7

These methods are simply the results obtained from methods 2–4 but the mean GL values were multiplied by the area of the DG (determined by outlining or thresholding), and these are referred to as the integrated grey level (IGL) values.

### Converting grey levels to optical densities

Data from the first method (line method) were then converted into three different units. The first two equations converted the GL values into optical densities (Eq. 2 and Eq. 4), and the third method used a ^14^C standard to convert GL values into units of radioactivity (Eq. 5).

The values obtained from the digital images are grey levels, which range from 0–255 for an 8-bit (2^8 ^= 256) image [25]. A grey level of 0 = white and 255 = black throughout this paper; the choice is arbitrary but it makes sense for the darker parts of an image to have higher values, as this corresponds to greater gene expression. Some of the equations below have been modified if the equations in the original paper used the reversed values, or if they used a scale ranging from 1 to 256 instead of 0 to 255. It should be stressed that the values obtained from an image are grey levels and not optical densities, which can be calculated in a variety of ways. Grey levels are often confused with optical densities (some imaging systems can be calibrated beforehand to output values as ODs rather than grey levels, but it makes no difference to the present discusion when the conversion takes place). The optical density of a sample is related to the amount of light that the sample absorbs (or alternatively, the amount of light it transmits), and for many applications (e.g. solutions and gases) is defined as:

(1) $$OD=log_{10}\left(\frac{I_{0}}{I_{1}}\right)\cdot \frac{1}{\ell }$$

where *I*_0 _is the intensity of the incident light (on the sample), *I*_1 _is the intensity of the transmitted light (through the sample), and ℓ is the distance that the light travels through the sample. Equation 1 is used to calculate the optical density of solutions or gases, but is modified for the analysis of gene expression by ISH; for example, the thickness of the film (or the sections) are not taken into account and therefore the length term (ℓ) is removed. In addition, the value of the incident light is typically not measured, but the closest thing would be to measure the developed film in a location without any brain sections on it (this could be thought of as the 'blank'), although this seems to be rarely done. Furthermore, the transmitted light is not measured directly, but captured by a digital camera as a greyscale image.

There are a number of published methods that have been used to convert grey level values to optical densities. The first is a relative (uncalibrated) optical density (ROD), and is simply a nonlinear transformation of GL values to OD values without the use of a standard (Eq. 2; [13,26]).

(2) $$ROD=log_{10}\left(\frac{255}{255-GL}\right)$$

Another uncalibrated method is suggested by Rieux et al., although this method was used to analyse protein expression in tissues and not autoradiographic films [17]. With this equation, the mean grey level of the structure of interest is divided by the mean grey level of 'a reference region of maximal transmittance' (*GL*(*max*); Eq. 3). Rieux et al. did not mention which region this was, but presumably either a region of tissue that does not express the gene of interest or an area of the film with no tissue on it. If the film with no sample on it is used as the reference region of maximal transmittance, then this would be dividing by a constant and therefore has a similar form to the uncalibrated ROD (Eq. 2).

(3) $$OD=log_{10}\left(\frac{GL(object)}{GL(max)}\right)$$

A third method converts GL values into ODs by calibrating against a standard of known values using an optical density step tablet (available at ) and the four-parameter (*a*, *b*, *c*, *d*) Rodbard equation (Eq. 4).

(4) $$OD=d+\frac{\left(a-d\right)}{\left(1+{\left(GL/c\right)}^{b}\right)}$$

A final method involves calibrating against a radioactive ^14^C standard by exposing the radioactive standard to the film along with the samples containing the radioactive probe. The grey levels of the eight strips on the standard are then measured and compared to radioactivity values provided by the manufacturer. An exponential model is then fit to the data and used to convert sample GLs to units of radioactivity using equation 5. However, other equations such as third and fourth degree polynomials have also been used [13]. *RA *is radioactivity in nCi/g, and *a *is the slope determined from the ^14^C standard.

(5) *RA *= *e*^*a*·*GL*^

It should be noted that some studies first convert GL values to ODs and then into units of radioactivity, so they are not mutually exclusive options [27].

### Statistical analysis

Analysis and simulations were conducted with R (version 2.8.0; [28,29]). The coefficient of variation was used to determine the precision of the various methods and a nested random-effects model was used to calculate the variance components [30-32]. A linear model of the GL values (*y*) can defined as

(6) *y*_*ijk *_= *μ *+ *R*_*i *_+ *S*_*ij *_+ *ε*_*ijk*_

where *μ *is the grand mean, *R*_*i *_is the difference of the *i*^*th *^rat from the grand mean, *S*_*ij *_is the difference of the *j*^*th *^section from the average for that rat, and *ε*_*ijk *_are the residuals. Associated with each level are the variance components; $\sigma_{R}^{2}$ is the variability of rats about the grand mean, $\sigma_{S}^{2}$ is the variability of sections nested within rats, and $\sigma_{\varepsilon }^{2}$ is the residual term which is the variability of the left and right side within sections (plus measurement error). The total variability ($\sigma_{T}^{2}$) is simply the sum of the three variability values and therefore the percentage of variability at each level can be calculated. The raw data are provided [Additional File 1] along with the R code [Additional File 2].

## Results

### Precision of different segmentation methods

**Figure 2:**
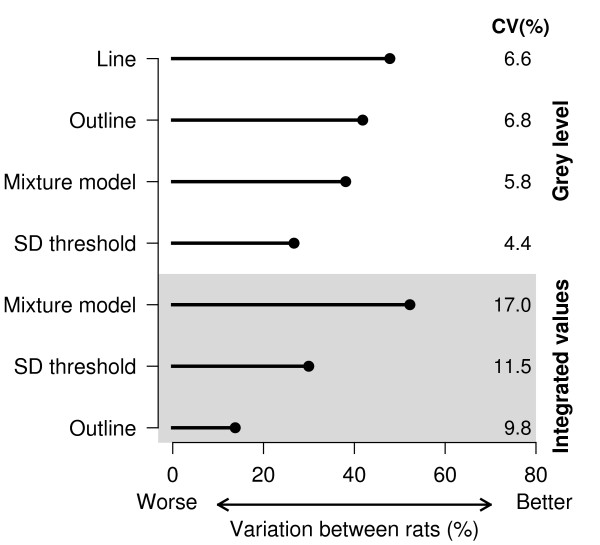
**Coefficients of variation and variance components analysis**. The coefficients of variation are larger for the integrated grey level values than their non-integrated counterparts. This is due to the extra noise added when the GL value is multiplied by the area of the structure, which reduces the precision and statistical power of subsequent analyses. The two thresholding methods had the lowest CVs, but this may be due to a floor-effect. The line method captured the most biological variation (between rats) compared to the other non-integrated methods, although the outline and the mixture model method performed similarly.

**Figure 3:**
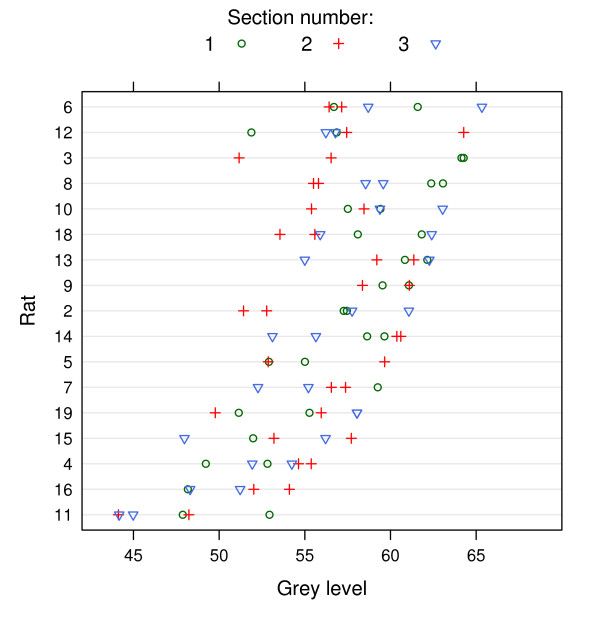
**Variability of grey level values at different levels of the sampling hierarchy**. Grey level values on each of three sections per rat are plotted. Values on the same section (left and right side) are plotted with the same symbol, and different sections are plotted with different symbols. Rats are sorted top to bottom from highest to lowest mean GL value, which allows the variability between rats to be compared to the variability within rats. The variance components analysis calculates the amount of variability at each level of the sampling hierarchy. The line method had the lowest amount of variability within and between sections and therefore the greatest variability was between rats, that is, it gave the most consistent results for multiple measurements on the same rat.

Mineralocorticoid receptor expression in the dentate gyrus was quantified using the line, outline, SD threshold, and mixture model method of segmentation (Methods 1–4) and the integrated methods (Methods 5–7) in order to assess their precision. Precise methods are preferred because they increase the statistical power of subsequent analyses, making it more likely that true differences between groups will be detected [33]. The coefficient of variation (CV) was calculated by dividing the standard deviation of each method by the mean of that method, and multiplying by 100. The CV measures the amount of variability, taking into account that numbers with a high mean value typically have greater variability. The four non-integrated methods had similar CVs (4.4 to 6.6), suggesting that the variability of the four methods, relative to their mean values, were similar (i.e. similar precision; Fig 2). However, when the area of the DG was included in the calculation, the CVs more than doubled, leading to a loss of precision (note that there is no area associated with the line method and therefore there is no integrated value). This result is not surprising, since the variability in the area of the DG (a combination of biological variability and measurement error) is now included in the final value, resulting in greater noise. Another method of examining variation in the data is with variance components analysis [[34], p. 638–640]. The data in this experiment were sampled at three different levels: (1) within sections (left and right side), (2) between sections (there are three sections per animal), and (3) between animals. The CV compares variability between animals at the highest level, but it is also possible to determine how the variability is distributed among the different levels. Ideally, one would like to have low within and between section variability (i.e. similar values for the left and right side of the brain and between each section for a particular animal), with most of the variability between different animals ($\sigma_{R}^{2}$). Figure 3 plots the values for each rat, showing the variability within sections (plotted with the same symbol), between sections (different symbols), and between rats (on separate lines). Section one for each rat is the most rostral while section three is the most caudal. There does not appear to be a rostral-caudal gradient of MR expression, as any section can have the highest or lowest value for a particular rat, and therefore the multiple sections can be thought of as replicate measurements used to better estimate the true value of MR expression for each rat. The results of the variance components analysis are plotted in Figure 2, where the methods are sorted (top to bottom) according to the amount of between-rat variability. The line method has the highest between-rat variance when using GL values, and only the integrated mixture model method had a higher value (although this method had the highest CV and therefore the lowest precision). The important finding is that multiplying GL values by an area measurement generally increases the overall variability in the data and thus is not recommended. The two thresholding methods had the lowest CV and might appear to be good methods, but this may be due to a floor-effect, where pixels with low values are not included, thus reducing the range of the data (see below).

### Integrated values have the potential for bias

Including the area of the structure or region of interest as an integrated GL value has the potential to bias the results because the ingreated value is a product of both the GL (i.e. amount of gene expression) and the size of the structure. Similar levels of gene expression between groups or conditions could be mistakenly concluded as being different if the size of the underlying structure differs between groups; differences in the IGL would reflect differences in the area, and not in gene expression. This is a well-known problem in the stereological literature and the reason why modern stereological methods do not include the area of the structure or region of interest when determining the total number of objects (e.g. cells, synapses, etc. [35-37]). Based solely on a reported integrated GL, there is no way to know whether any significant differences are due to differential gene expression or simply differences in the size of the structure. Therefore, at best, including the area in an integrated value simply increases the variability of the data; at worst, it can bias the results, and therefore should be avoided.

### Potential floor-effect with thresholding methods

**Figure 4:**
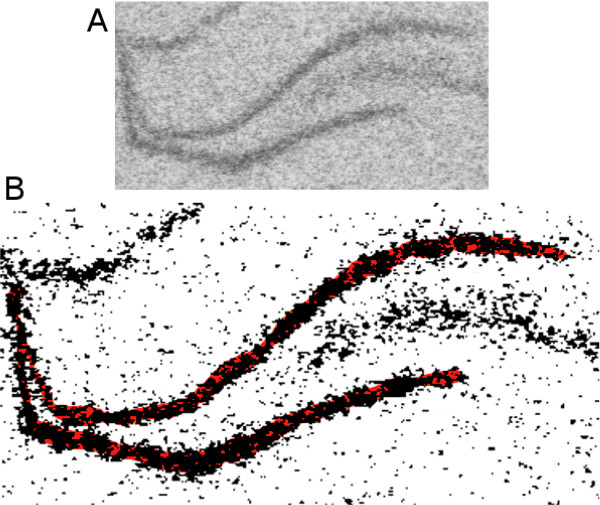
**Thresholding floor-effect**. The dentate gyrus (A) was thresholded and the selected pixels are displayed in black (B). There are pixels that are a part of the DG (indicated in red) that should be included in the calculation of the mean GL, but have fallen below the threshold level and are therefore excluded. This biases the GL value upward, and creates a floor-effect, where no pixels below the threshold are included in the calculation of the mean GL.

**Figure 5:**
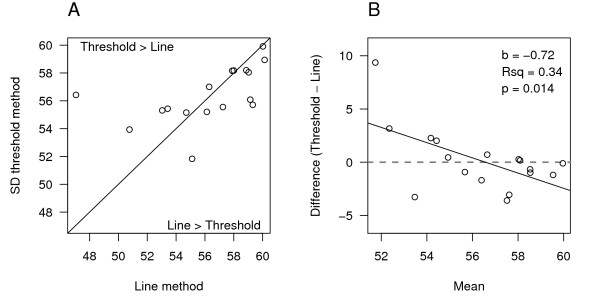
**Detection of a floor-effect**. The average values for each rat obtained from the thresholding method (Method 3) and the line method (Method 1) are plotted (A). The black line is the line of identity (*y *= *x*) and points above the line are those for which the threshold method had larger values. A mean-difference plot is shown in (B), where values on the *y*-axis are the difference between the threshold and line methods, and on the *x*-axis the mean of the two methods. Points above the horizontal grey line had larger values with the threshold method, while points below the line had larger values with the line method. The significant slope (*b*) indicates that the threshold method gives higher values then the line method at lower GL values, which is what would be predicted if there is a floor-effect.

The SD thresholding method selects the foreground as being above a certain level of the background, and the mixture model partitions the pixels into one of two Gaussian distributions (one foreground and the other background). A major disadvantage of these approaches is that spatial information is not included when distinguishing the foreground from the background, only the GL value of each pixel. Thus, when using thresholds to select the structure–especially when gene expression in the structure or region of interest is low relative to background levels–parts of the structure might be omitted, where pixels with GL values lower than the threshold are excluded from the calculation of the mean GL value, even though they are in the structure of interest and should therefore be included. This has the potential to bias the results upwards and can be seen in Figure 4, where parts of the dentate gyrus (in red) are excluded from the analysis. An analogy is with trying to determine the mean size of fish in a lake by casting a net into the water; fish that are smaller than the holes in the net will slip through and will not be included in the calculation of the mean, resulting in a higher estimated mean value than the true population value. The lighter coloured pixels below the threshold but in the DG are analogous to the smaller fish that slip through the net. This is the likely explanation for the low CV of the two thresholding methods (Fig. 2); the range of values is restricted because none can be lower than the threshold. This was examined directly by plotting the values for the line (Method 1) and SD thresholding method (Method 3) against each other (Fig. 5A). The diagonal line is not a regression line, but the line of identity (*y *= *x*) and points above the line are those for which the threshold method gave greater values than the line method, and the opposite for points below the line. A Tukey mean-difference plot was used to better examine the  relationship between the two methods (Fig. 5B, [38,39]), where the difference between the threshold method and line method is plotted on the *y*-axis and the mean of the two methods is plotted on the *x*-axis. Similar to the previous graph, values above the horizontal *y *= 0 line (grey) are the ones for which the threshold method gave the larger value, and values below the line are ones where the line method gave the larger value. Ideally, the points would fall along the *y *= 0 line, indicating that on average the two methods give the same values. Alternatively, if there was an additive shift of say five units above the *y *= 0 line, this would represent the threshold method consistently giving higher values than the line method. Based on this, we cannot determine if the threshold method is an overestimate or if the line method is an underestimate, but given the semiquantitative nature of the technique, such a result would not pose any problems for analysis or interpretation. However, when there is a trend in the values on the mean-difference plot, it indicates that the two methods produce different results at different levels of pixel intensity. In this case the threshold method has higher values at lower GL values, indicating that the threshold method values do not decrease as quickly as the line method values at lower GL values, consistent with with a floor-effect (*p *= 0.014). While the trend is relatively small with this data, this is a serious limitation of thresholding methods and they should therefore be avoided. It would not be apparent if a floor-effect is present in a dataset unless the results are compared with another method.

### Converting grey levels to other units

**Figure 6:**
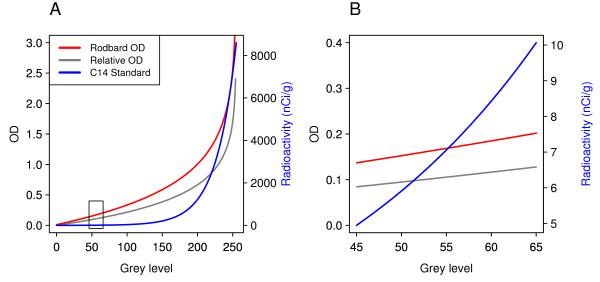
**Transformation of grey levels**. Grey levels from 0 to 255 were transformed using three different methods (A). The uncalibrated relative OD and the Rodbard OD method are plotted on the left *y*-axis, while transformation with the ^14^C-standard uses the right y-axis. The box indicates the range of observed grey level values in this dataset and are re-plotted in (B). All three methods give different curves, however within the range of actual values, the transformation is fairly linear, especially for the relative and Rodbard OD transforms.

**Figure 7:**
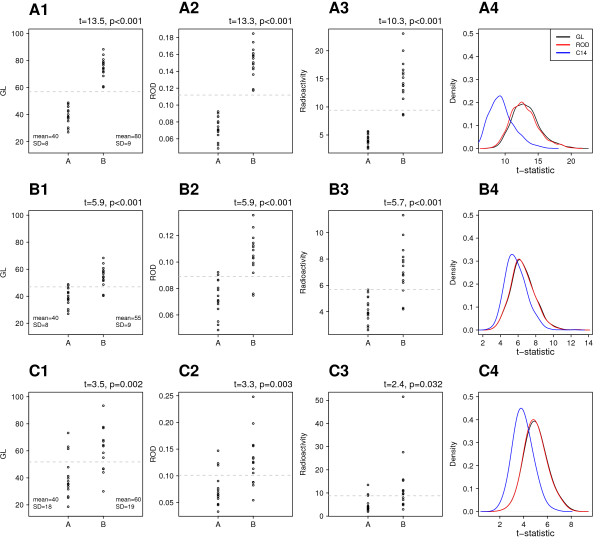
**Effect of transformations on simulated data**. Data in the first column are the simulated grey level values from various distributions. Note that the *y*-axis is the same in these three panels so the data can be compared directly. A1 has a large difference between means and low variability, leading to no overlap between data points. B1 has similar variability as A1 but the difference in means has been decreased by half, leading to overlapping values between groups. C1 has the same difference in means as B1, but much greater variability. Horizontal grey lines are the grand mean and can be used as a visual guide to compare the distribution of data points under various transformations (across rows). Converting to ROD has little effect on the statistical properties of the data, while converting to units of radioactivity badly skewed the data, created outliers under certain conditions, and leads to decreased power (see text for details). This is highlighted in column four where the *t*-values for the radioactivity data (blue line) are shifted to the left for all three datasets.

It is common for grey levels to be converted into optical densities or expressed as units of radioactivity. The purpose of these conversions is ostensibly to account for the nonlinear relationship between the transmittance of light through the film (Beer's Law) and for the nonlinear relationship between the darkness of the film and the number of particles striking the film from radioactive decay. Because these transformations are nonlinear, they have the effect of making high values in the data disproportionately higher. Figure 6 displays the effect of transforming GL values into relative optical densities (Eq. 2), calibrated optical densities (Eq. 4) or calibrated units of radioactivity (Eq. 5). While these are nonlinear transformations, the range of the observed experimental values in the present study was narrow compared to the range of possible values (approximately 8% of the range). This will likely be true for many studies, where the GL values between conditions will be within a fairly narrow range. If this is the case, then transforming the values is pointless (it is the equivalent of converting from degrees Celsius to degrees Fahrenheit, and performing statistical analysis on the converted data); and if the GL values have a wider range, then such transformations skew the distribution and/or create outliers as demonstrated below. In order to assess the effect of these transformations on the statistical properties of the data, three datasets with different characteristics were simulated, and the results displayed in Figure 7. Data for two groups (A and B) were drawn from normal distributions with *n *= 15 in each group; the parameters of the distributions are shown in the figure. The two groups were analysed with a two-tailed independent samples t-test with Welch's correction for unequal variances [40]. The *t*-value provides a useful metric to compare the effect of various transformations, as it reflects the differences between the means of the groups divided by the variability (note that Welch's correction adjusts the degrees of freedom and not the test statistic). The A-series data was constructed to have a large difference between the means of the two groups and low variability in each group, such that there is no overlap in the distributions. In such a case statistical inference is hardly necessary and *t *= 13.5. Transforming the values to ROD (Eq.2; A2) does little in the way of changing the result of the statistical analysis (*t *= 11.3), or one's subjective impression of the plotted data. Converting to units of radioactivity however (Eq. 5; A3) skewed the distribution and made the variance of B sixteen times bigger than A (Fligner-Killeen test: $\chi_{1}^{2}$ = 8.1, *p *= 0.004). This is to be expected of such nonlinear transformations where the original grey level values cover a wide range. While visually it may seem that group A and B are now 'more different', the *t*-statistic has become much smaller (*t *= 10.3) due to the increased variability, indicating a smaller effect. One thousand datasets were randomly generated with the above parameters and the *t*-statistic was calculated for each. The results are plotted in panel A4 where it can be seen that the distribution of *t*-statistics does not change when converting to ROD, but are shifted to the left when transforming grey levels to units of radioactivity, indicating reduced power of any statistical analysis. All of these would still be significant, as the t-statistics are large, but it makes the point that such transformations can reduce the power of subsequent analyses. Given that the group with the higher mean has the higher variance in A3, it is common to deal with this type of data by log transformation. The irony is that this reverses the operation of converting GL values to units of radioactivity, which was an exponential transformation. Alternatively, a non-parametric test could be used (e.g. Wilcoxon/Mann-Whitney), but the results would be identical for all three (GL, ROD, radioactivity), because the analysis is done on the ranks and not on the raw values, and all of these transformations will have no effect on the rank ordering of the data. With such data it would appear that performing the analysis on the GL values is perferable.

The second B-series data has the same variability as the A-series but the difference in means has been halved, such that the distributions now overlap. This also means that the total range of the data has been reduced, and this likely represents a more common arrangement of data that would be obtained from real experiments. Similar to the previous data, converting to ROD (B2) does little to alter the subjective interpretation of the results upon viewing the graph, and there is little or no change in the statistical properties of the data (B4). Converting to units of radioactivity (B3) appears to have increased the difference in the means of the two groups, but this is offset by increased variability, which leads to a decrease in statistical power (smaller *t*-value), and is again indicated by a shift to lower *t*-statistics (B4). Finally, data in panel C1 has the same difference between means as B1, but increased variability (and increased range of data). Once again, converting to ROD (C2) changes the statistical properties little, and converting to units of radioactivity has increased the variance in group B and created an outlier. Again, such data might be suitable for log-transformation to normalise the variances, or a decision has to be made whether to remove the offending point. When both groups have similar means and variances, neither transformation affects the *t*-statistic (not shown).

This analysis tells us, first, that if the data are in a narrow range (e.g. B-series) then the transformation to ROD is linear and the transformation to units of radioactivity fairly linear, with a small decrease in power due to increased variability. Second, if the data cover a wide range of values–either because the means of the groups are far apart or due to high variability–then the transformation to units of radioactivity will create outliers, skewed distributions, or both, thus creating problems for subsequent statistical analysis. The conclusion is that for the majority of studies it is better to analyse the GL values directly rather than convert them to other units. This also has the advantage of fewer calculations (less chance for computational errors), it is faster, and the data can be related to more intuitively; for example, possible values range from 0 to 255, and 80 is twice as high as 40. This does not mean that gene expression is twice as high however, but given that autoradiography is a semiquantitative technique, GL values are not directly related to the number of mRNA transcripts. It is more difficult to intuitively compare two values on a multiplicative scale that have been nonlinearly transformed.

## Discussion

A variety of methods have been used in published studies for image segmentation (manual outlining, thresholding, magic wand, use of templates, etc.) to determine what is part of the structure or region of interest and what is not. Similarly, once grey levels are measured, a variety of methods have been employed to convert them into optical densities or units of radioactivity. Based on the above results, the line method was the best way to select the structure or region of interest as it was not subject to floor-effects, had a low coefficient of variation, and low within-sample variability. This method is similar to, and a modification of the outline method, which requires that the actual boundaries of the structure be determined, whereas the line method sampled only from the interior of the structure. Since the DG is a long narrow structure, this was best done with a line down the centre. Larger structures can follow the same principle by 'outlining' the structure but staying well inside the border so that only the interior is sampled. A drawback of trying to outline the border of the structure is that it is not always clear exactly where it lies, especially when gene expression is low relative to background levels. A second drawback is that the need for hand-eye-mouse coordination can introduce some additional variability, although this was relatively mild with the present data as both the line and outline method had similar coefficients of variation and within-sample variability. This method is also relatively fast since structures do not have to be carefully delineated, and the only calculation involves background subtraction. Thresholding methods are common, but as was shown here, can be subject to a floor-effect, limiting their usefulness in many cases. These results may not apply to quantifying gene expression in the neocortex, where due it its laminar structure, it is common to use transects to determine gene expression across the different layers [41].

Once the structure is selected, only the GL value and not the integrated GL value should be used for further analysis. Using integrated GL values will be rarely preferable for analysis of films because changes in the size of the underlying structure and not changes in gene expression may be affecting the results. Changes in area could then be misinterpreted as changes in gene expression. However, even if areas are similar between groups or conditions, including the area increases the variability of the data, thereby decreasing precision and statistical power. The area should be reported separately (as in reference [19]), if at all, and the area of the actual structure obtained from the histological sections should then be measured and reported as well (alternatively, the volume of the relevant structure could also be reported). This is easily done as slides can be counter-stained with Cresyl violet after exposing the film, and the area determined. Integrated grey levels are appropriate for analysis of gels (e.g. Western blot, dot blot) because the protein, DNA, or RNA are not bound within cells and within structures as they are *in vivo*. Both the darkness and the area are then needed to quantify the amount of substance present, as the mean grey level of the dot or band will decrease as the substance is spread out over a larger area–this does not apply to histological sections.

Finally, statistical analysis should be performed on the untransformed GL values (averaging across sections to give one value per animal). There appears to be little advantage to transforming GL values into either optical densities or units of radioactivity. Some may argue that the relationship between the amount of radioactivity and the response of the film is nonlinear, and that the imaging system's response to levels of darkness are also nonlinear, and that these need to be adjusted somehow, e.g. by converting to ROD and then using ^14^C standards. However, one must bear in mind that autoradiography is only semiquantitative, which means for example, that while the difference in GL values between 40 and 35, and 50 and 45 is five, it does not necessarily represent an equivalent change in the number of mRNA transcripts. Adjusting for such small non-linearities suggests a much higher quality of data than is actually obtained with ISH and autoradiography. Furthermore, the grey level values will likely be in a narrow range in most studies, which means these transformations are linear and therefore pointless (as in converting from Celsius to Fahrenheit). Alternatively, if the data have a wide range, then such transformations can result in a combination of (1) increased overall variability, (2) heterogeneous variances between groups, and (3) outliers, resulting in a reduction in statistical power, and necessitating log transformations or non-parametric tests, which either reverses (log transformation) or ignores (rank-based statistical tests) the effect of such transformations. Grey levels obtained directly from the imaging system are suitable values to use for analysis and no further calculations are required. Other advantages include less time to carry out the analysis, less chance for computational errors, the values are easy to interpret (i.e. they range from 0–255 for and 8-bit image), and easy to compare between studies–it is often a mystery what the values on the *y*-axis of graphs represent in many studies.

There is one instance when using a ^14^C standard is useful: when multiple films are required because not all samples can fit on a single film. There will likely be systematic differences between films, and the ^14^C serves as a common reference that allows direct comparisons of samples from multiple films. There are however other alternatives such as converting the results within each film to *z*-scores, which standardise the data within a film to have a mean of zero and a standard deviation of one. The *z*-scores can then be analysed in the normal way. This requires that brains from different experimental conditions are (approximately) balanced across the films–this means not having all the controls on one film and all the treated animals on an other film. This should already be standard practice and so does not introduce any additional procedures or constraints, and it has the advantage of (1) using only a linear transformation and (2) not requiring anything else to be estimated and incorporated into an equation, which will almost certainly introduce more noise. The present study used only one film and so it was not possible to assess the relative merits of using ^14^C standards versus *z*-scores.

While this study only examined gene expression in one brain region, it is likely that most of the results apply to other regions and structures as well, although the extent to which such concerns as bias and reduced precision play a role outside of the hippocampus will have to be empirically determined. The data and R code are therefore provided so that readers can reproduce the results of this paper and use them as a template for the analysis of their own data.

## Conclusion

Based on the above results, three recommendations are proposed. First, do not use integrated values because they are a function of both the mean grey level (i.e. gene expression) and area, making the results difficult to interpret; bias can be introduced if the area of one group is different than another group (even though GL values are the same). In addition, integrated values have reduced precision because the variability in the estimation of the area is included in the final value. Areas can be reported separately if required, although this arguably provides little in the way of new information and it is preferable to estimate area on tissue sections directly rather than on autoradiographic films. Second, manual selection of the interior of the structure or region of interest results in data with low variability (low CV), avoids ambiguities in determining the edge of structure, and is a relatively quick method requiring few calculations (only background subtraction). However, the standard method of outlining the structure by hand proved to have suitable properties with this dataset as well. Given the possibility of floor-effects with thresholding methods (at least with the global methods examined in this paper), they should be avoided. Third, statistical analysis should be performed on the GL values without transforming them to optical densities or standardising them against ^14^C standards (unless multiple films are used). The dynamic range of images in most studies will be fairly narrow and therefore these transformations are pointless. If the range of the data is large, standardising the values against ^14^C standards can have negative consequences for the distributions (skewness, outliers, and heterogeneous variances).

In summary, several suggestions have been made which should be employed in the analysis of gene expression on autoradiographic images to reduce bias, increase precision, and ultimately provide more meaningful results.

## Supplementary Material

Additional File 1 **Data file**. The raw data are provided as a CSV file. Click here for file

Additional File 2 **R functions**. The R code to produce some of the figures and analyses are provided as a plain text file. Click here for file
